# Supplementary material for: Content-rich biological network constructed by mining PubMed abstracts
Source: BMC Bioinformatics. 2004 Oct 8;5:147. doi: 10.1186/1471-2105-5-147 (PMC528731; doi:10.1186/1471-2105-5-147)
Supplement: Additional File 2 — The original results of the above study (non-essential files are deleted to keep the file size under the limit set by BMC bioinformatics). [file 1471-2105-5-147-S2.bz2 › chilibotAdditionalFile2/dip30/492ID7706250E10655/html/TGFB1_TGFBR1.html]

 


 **TGFB1** and **TGFBR1** 
  
Found 188 abstracts in PubMed, retrieved 30.  
 

 What does Google say? 
 PDF only 
| .edu only 

---

**Interactive relationship** (e.g. stimulation, inhibition, etc)

**Neutral relationship**- Activin receptor like kinase 5  [ **TGFBR1** ]  ALK 5  [ **TGFBR1** ]  is a TGF beta  [ **TGFB1** ]  type I receptor expressed in various TGF beta  [ **TGFB1** ]  responsive cells.  Ref: 12384983 J Cell Physiol, 2002
- ALK 5  [ **TGFBR1** ]  and ALK 1 mediate TGF beta  [ **TGFB1** ]  signals through distinct Smad proteins, i.e., Smad2 Smad3 and Smad1 Smad5, respectively.  Ref: 12384983 J Cell Physiol, 2002
- In addition three of the residues required for activin binding to ALK4 are conserved on the type I TGF beta  [ **TGFB1** ]  receptor ALK5  [ **TGFBR1** ] , suggesting the corresponding region on ALK5  [ **TGFBR1** ]  may be important for TGF beta  [ **TGFB1** ]  binding  Ref: 12665502 J Biol Chem< MedlineTA>J Biol Chem, 2002
- In endothelial cells, TGF beta  [ **TGFB1** ]  binds to two distinct type I receptor serine threonine kinases, ALK 5  [ **TGFBR1** ]  and ALK 1.  Ref: 12297674 Sci STKE, 2002
- These results suggest that TGF beta 1  [ **TGFB1** ]  regulates two key dentin proteins involved in matrix mineralization most likely mediated through the type I ALK5  [ **TGFBR1** ]  receptor and transduced by Smads, and 4.  Ref: 12489180 Connect Tissue Res, 2002
- These genes were TGF beta  [ **TGFB1** ]  type I receptor gene  **TGFBR1** , TGF beta  [ **TGFB1** ]  type II receptor gene TGFBR2, SMAD2 gene SMAD2, SMAD3 gene SMAD3, SMAD4 gene SMAD4, and SMAD7 gene SMAD7, all of which compose the TGF beta1  [ **TGFB1** ]  signaling pathway.  Ref: 12202987 J Hum Genet, 2002

**Non-interactive relationship** (e.g. studied together, co-existance, homology, etc.)

- An inhibitor of the TGF beta  [ **TGFB1** ]  type I receptor ALK5  [ **TGFBR1** ]  was able to prevent the stimulation of alpha3 IV and VEGF proteins by HG.  Ref: 12164872 Kidney Int, 2002
- TGF beta1  [ **TGFB1** ] , ALK 1, ALK 5  [ **TGFBR1** ] , and TbetaRII were also detected by immunohistochemistry.  Ref: 12883979 Pediatr NephrolPediatr Nephrol, 2002
- Therefore, we generated TGF beta  [ **TGFB1** ]  type I receptors with mutations in the L45 loop of the kinase domain, termed ALK5  [ **TGFBR1** ]  and ALK5  [ **TGFBR1** ] .  Ref: 12446693 J Biol Chem, 2003
- Our findings indicate that the TGF beta 1  [ **TGFB1** ]  type I receptor ALK5  [ **TGFBR1** ]  is expressed by odontoblasts as well as the signal transduction proteins Smad2, Smad3, and Smad4.  Ref: 12489180 Connect Tissue Res, 2002
- Enalapril decreased renal TGF beta1  [ **TGFB1** ] , ALK 1, and ALK 5  [ **TGFBR1** ]  protein expression P< ; 0.05.  Ref: 12883979 Pediatr NephrolPediatr Nephrol, 2002
- These data demonstrate that an autocrine transforming growth factor beta1  [ **TGFB1** ]  ALK5  [ **TGFBR1** ]  pathway is a negative feedback mechanism for epidermal growth factor induced normal human keratinocyte growth.  Ref: 12787131 J Invest Dermatol, 2003
- Characterization of their signaling properties revealed that the two L45 loop mutants did not mediate Smad dependent transcriptional responses, TGF beta  [ **TGFB1** ]  induced growth inhibition, and fibronectin and plasminogen activator 1 production in R4 2 mink lung epithelial cells lacking functional ALK5  [ **TGFBR1** ]  protein.  Ref: 12446693 J Biol Chem, 2003
- Boston minipigs, treated with tranilast or vehicle, were subjected to endoluminal stenting, and the expression of TGF beta1  [ **TGFB1** ]  and TGF beta3, the expression of their signaling receptors ALK 5  [ **TGFBR1** ]  and TbetaR II, leukocyte numbers around the stent struts, and neointima development were assessed over 28 days.  Ref: 12067902 Arterioscler Thromb Vasc Biol, 2002
- This study was designed to investigate the relationship between renal growth impairment and the expression of transforming growth factor beta1  [ **TGFB1** ]  TGF beta1  [ **TGFB1** ] , TGF beta  [ **TGFB1** ]  receptor I TbetaRI, activin like kinase ALK 1 and ALK 5  [ **TGFBR1** ] , and TGF beta  [ **TGFB1** ]  receptor II TbetaRII.  Ref: 12883979 Pediatr NephrolPediatr Nephrol, 2002
- Newborn rat pups were treated with enalapril 30 mg kg per day or vehicle for 7 days, and kidneys were removed for Western blotting of TGF beta1  [ **TGFB1** ] , ALK 1, ALK 5  [ **TGFBR1** ] , and TbetaRII, and for RT PCR of ALK 5  [ **TGFBR1** ]  and TbetaRII.  Ref: 12883979 Pediatr NephrolPediatr Nephrol, 2002
- These results indicate that ACE inhibition in the developing kidney decreases TGF beta1  [ **TGFB1** ] , ALK 1, and ALK 5  [ **TGFBR1** ]  expression, which may account for renal growth impairment.  Ref: 12883979 Pediatr NephrolPediatr Nephrol, 2002
- These results revealed some new targets of TGF beta  [ **TGFB1** ]  in endothelial cells, and differences in transcriptional regulation patterns between ALK 1 and ALK 5  [ **TGFBR1** ] .  Ref: 12384983 J Cell Physiol, 2002
